# S2-1 Physical literacy in Europe

**DOI:** 10.1093/eurpub/ckad133.009

**Published:** 2023-09-11

**Authors:** Chair(s):, Johannes Carl, Peter Elsborg

**Affiliations:** Department of Sport Science and Sport, Friedrich-Alexander University Erlangen-Nürnberg, Germany; Copenhagen University Hospital – Bispebjerg and Frederiksberg, Center for Clinical Research and Prevention, Denmark

## Abstract

**Introduction:**

Most approaches in health-enhancing physical activity with a focus on person-related qualities take a fragmented view on physically, active lifestyles, as they concentrate on unidimensional constructs with an isolated perspective. However, physical literacy (PL) can be described as a holistic and integrative concept that simultaneously considers physical, cognitive, and affective determinants for physical activity. The PL concept has gained momentum in recent years and its promotion has been suggested as a key mechanism within the Global Action Plan on Physical Activity 2018-2030 (World Health Organization) for the reduction of worldwide physical inactivity in the future.

**Presentations:**

The present symposium starts with an introduction into the concept, including its history, philosophic underpinnings, and most important assumptions. The findings of the EUROPLIT study will frame the further course of this symposium, as this study comprehensively assessed and compared the situation of PL in research, practice, and policy across Europe. In this context, the present symposium will zoom into the specific situation of three European countries and exemplarily present studies that reflect the national developments of PL. In the first country-specific contribution, Portuguese researchers will report on the current state of PL assessments and PL interventions within their country. In the second country-specific contribution, a research team from different institutions will illustrate how the PL concept guides the design of interventions in Denmark and how these are implemented into practice and policy. In the third country-specific contribution, two Austrian researchers will describe the multi-phase development process of a PL questionnaire for adults in German language.

**Q&A:**

The symposium concludes with a discussion (moderated by the two chairs) on the most relevant blind sports in research, future pathways as well as consequences for practice and policy. In summary, the symposium bridges the gap between research, practice, and policy. Therefore, it can make a major contribution to this years’ conference focus.

